# Pilomatricoma in the neck of an adult male

**DOI:** 10.4322/acr.2021.379

**Published:** 2022-04-28

**Authors:** Kofi Ulzen-Appiah

**Affiliations:** 1 Cape Coast Teaching Hospital, Department of Pathology, Cape Coast, Ghana

**Keywords:** Pilomatrixoma, Child, Head, Head and Neck neoplasms, Neoplasms

Pilomatricoma (also spelt Pilomatrixoma and known as Calcifying epithelioma of Malherbe), was first described by Malherbe and Chenantais^1^ in 1880 as a calcified tumor, originating from sebaceous glands. Later, in 1961 Forbis and Helwig^2^ carried out histochemical and electron microscopic studies of pilomatricoma and deduced the cell of origin to be from the outer sheath cell of the hair follicle root. Pilomatricoma is often found in children and young adults, nonetheless, it can occur in any age group, with a higher incidence in females (female: male ratio 3:2).^3^ The majority of affected individuals are Caucasians.^3^ The tumor primarily affects children and adolescents with 40% of cases occurring before age 10 and 60% before age 20.^4^ The greatest incidence of this tumor is found between 8 and 13 years of age.

Clinically, the tumor usually appears as a firm solitary, painless, slow-growing nodule ranging from 0.5cm to 3cm in most cases.^5^^-^7 The tumor is freely mobile within the relating surrounding tissue structures and overlying skin. They are commonly located in the head and neck region, followed by the upper extremities, the trunk, and the lower extremities.^2^^,^^3^ The usual locations in the head and neck region, are the neck and the frontal, temporal, periorbital, and periauricular areas of the head.^6^^,^^7^

Differential diagnoses of head and neck pilomatricoma include dermoid, epidermoid, and branchial cleft cysts, periauricular sinuses, adenopathy, salivary gland tumors, giant cell tumor, chondroma cutis, necrotizing granulomatous lymphadenitis and foreign body reactions.^5^^-^7 Diagnosis of pilomatricoma can be made solely on the basis of histopathological features, typified by varying amounts of 2 components mixed together in a disorganized fashion in the dermis, that is anucleate pink “ghost” cells (sheets of dead keratinocytes in which the ghost/shadow outline of each individual cell can still be seen, and aggregates or sheets of small round blue cells representing a germinative/matrical epithelial component (similar to the round blue cells seen in the hair bulb/roots of normal hair follicles).^8^ Each tumor nodule is often surrounded by brisk granulomatous inflammation, fibrosis, and foreign body giant cell reaction to keratin of the anucleate ghost cells.^8^ Calcifications are often present and metaplastic bone formation can occur.^8^ Mitoses may be quite frequent in the round blue cell matrical component, but nuclear pleomorphism should not be seen.^8^ Brisk mitotic activity alone should not be regarded as evidence of malignancy.^8^

These tumors are typically solitary, but multiple tumors have been reported. Multiple pilomatricomas are seen in patients with Gardner syndrome (the extraintestinal variant of familial adenomatous polyposis)^9^ and as a cutaneous sign of myotonic dystrophy.^10^ Malignant transformation of pilomatricoma is rare,^6^ and the standard treatment is by complete surgical excision.^2^^,^^5^^-^7^,^^11^ Recurrence of this tumor is rare after excision, with an incidence of 0-3%.^2^^,^^5^^-^7^,^^11^

The images sum up a 22-year-old man who presented with a right lateral subcutaneous neck nodule, which was not affixed to overlying skin and underlying related tissue structures. The mass was excised and submitted to histopathological examination.

**Figure 1:**
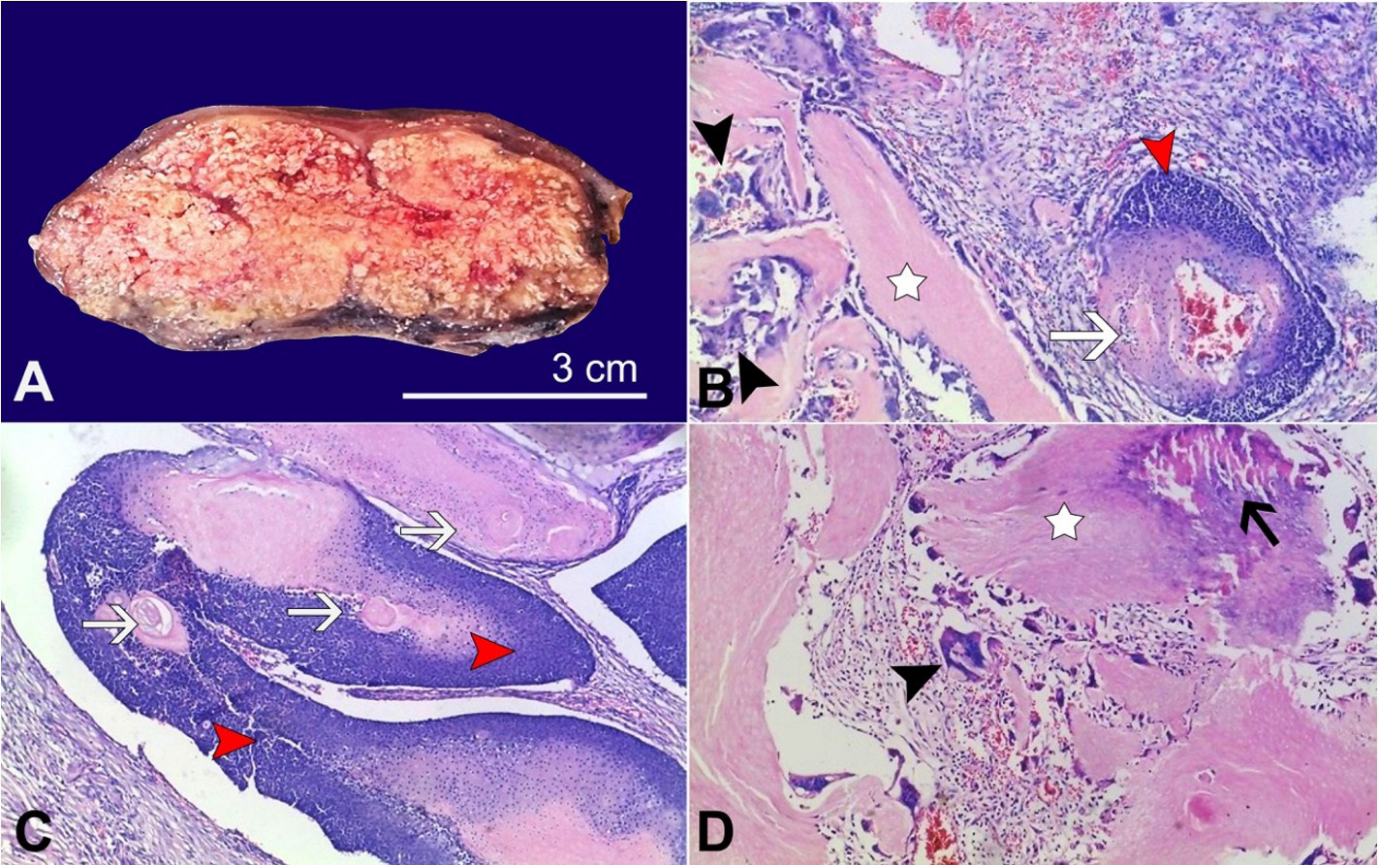
**A –** macroscopic view of the cut surface of the nodule, disclosing a well-circumscribed firm chalky appearance; **B** and **C –** photomicrographs showing biphasic anucleate pink ghost cells (white star), lined by foreign body type giant cells (black arrowhead) admixed with islands of matrical/germinative epithelial component (small round blue cells) (red arrowhead) with abrupt trichilemmal type keratinization(white arrows) (H&E 40X); **D –** Photomicrograph showing islands of anucleate ghost cells (white star) with associated foreign body type giant cells (black arrowhead). Focus of calcification is present (black arrow) (H&E 40X).

Gross examination revealed a 200g nodule with a normal overlying ellipse of skin measuring 7x5x3cm and 9x6cm, respectively. Sectioning the mass disclosed a well-circumscribed firm grey-white chalky lesion measuring 6.5x3cm (Figure 1A). Histopathological examination of the lesion showed biphasic dermal lesion composed of islands of anucleate “ghost” cells (Figure 1B and 1C) and islands of small round blue cells (Figure 1B and 1C) with abrupt trichilemmal type keratinization (Figure 1C). In areas, the islands of anucleate ghost cells are surrounded by foreign body type giant cells in response to keratin of the ghost cells (Figure 1B, and 1D). Elsewhere calcification is present (Figure 1D). Based on these classic features, a diagnosis of Pilomatricoma was made.

The post-operative period was uneventful and at the last follow-up, no recurrence of the nodule was noted.
